# Human Papillomaviruses Preferentially Recruit DNA Repair Factors to Viral Genomes for Rapid Repair and Amplification

**DOI:** 10.1128/mBio.00064-18

**Published:** 2018-02-13

**Authors:** Kavi Mehta, Laimonis Laimins

**Affiliations:** a Department of Microbiology-Immunology, Northwestern University Feinberg School of Medicine, Chicago, Illinois, USA; University of Michigan—Ann Arbor

**Keywords:** ATM, ATR, BRCA1, DNA damage response, double-strand breaks, H2AX, HPV, RAD51

## Abstract

High-risk human papillomaviruses (HPVs) activate the ataxia telangiectasia mutated-dependent (ATM) DNA damage response as well as the ataxia telangiectasia mutated-dependent DNA-related (ATR) pathway in the absence of external DNA damaging agents for differentiation-dependent genome amplification. Through the use of comet assays and pulsed-field gel electrophoresis, our studies showed that these pathways are activated in response to DNA breaks induced by the viral proteins E6 and E7 alone and independently of viral replication. The majority of these virally induced DNA breaks are present in cellular DNAs and only minimally in HPV episomes. Treatment of HPV-positive cells with inhibitors of both ATM and ATR leads to the generation of DNA breaks and the fragmentation of viral episomes, indicating that DNA breaks are introduced into HPV genomes. These breaks, however, are rapidly repaired through the preferential recruitment of homologous recombination repair enzymes, such as RAD51 and BRCA1, to viral genomes at the expense of cellular DNAs. When HPV-positive cells are treated with hydroxyurea, this recruitment of RAD51 and BRCA1 to viral genomes is greatly enhanced with little recruitment to damaged cellular DNAs and with retention of the ability of viral genomes to amplify. Overall, our studies demonstrated that human papillomaviruses induce breaks into cellular and viral DNAs and that the preferential repair of these lesions in viral episomes leads to genome amplification.

## IMPORTANCE

High-risk human papillomaviruses (HPVs) are the etiologic agents of cervical cancer and are linked to the development of many other anogenital and oropharyngeal cancers. Replication of high-risk HPVs requires the activation of the ataxia telangiectasia-mutated (ATM) and ATM- and Rad3-related (ATR) DNA repair pathways. Our studies have shown that HPVs activate these pathways by inducing double-strand breaks primarily in cellular DNAs and minimally in viral genomes. Breaks are induced in viral genomes but are rapidly repaired through the preferential recruitment of homologous repair factors such as RAD51 and BRCA1 to HPV episomes. The preferential repair of breaks in viral genomes leads to amplification. Our study identified a novel mechanism by which human papillomaviruses manipulate DNA repair pathways to productively replicate viral genomes. The induction of genetic instability in cellular DNAs likely contributes to the generation of mutations that lead to the development of cancers.

## INTRODUCTION

Human papillomaviruses (HPVs) are small, nonenveloped double-stranded DNA viruses that infect stratified epithelia and link their life cycles to cellular differentiation. A subset of about nine HPV types (including HPV 31, 18, and 16) are referred to as high-risk strains and are the etiologic agents of 99.9% of cervical cancers as well as many oropharygeal cancers (1). HPV virions infect cells in the basal layer of stratified squamous epithelia and establish their genomes as low-copy-number nuclear extrachromosomal elements or episomes that are stably maintained for extended periods. In these cells, viral replication occurs coordinately with that of cellular DNAs, and replicated genomes are distributed equally to each of the two daughter cells. Following cell division, one daughter cell moves into suprabasal layers and begins to differentiate, which leads to late promoter activation and productive viral replication, which is referred to as amplification (1, 2). Differentiation-associated viral amplification has been shown to depend on the activation of the ataxia telangiectasia mutated-dependent (ATM) DNA damage response as well as the ataxia telangiectasia mutated-dependent (ATR) DNA-related pathway, but how these repair pathways become activated during viral infections is unclear (3–6).

The ATM-dependent DNA damage response (DDR) is required for the maintenance of host genomic integrity and stability. A cell develops DNA lesions that are repaired by either nonhomologous end joining (NHEJ) or homologous recombination (HR) (7). The most cytotoxic of these lesions are double-strand breaks (DSBs), and when these breaks cannot be repaired, cells undergo apoptosis. In the case of HR repair, DSBs are first sensed by the tripartite MRN complex, made up of MRE11, RAD50, and NBS1, which induces the autophosphorylation of the ATM kinase (8). Phosphorylation of ATM then leads to activation, phosphorylation, and amplification of a cascade of downstream effector proteins that include the modified histone H2AX, the kinase CHK2, and the cohesin protein SMC1 (9). These factors colocalize to distinct nuclear repair foci. Additional repair proteins such as 53BP1, BRCA1/2, NBS1, and RAD51 are also recruited to these sites, leading to DNA resection and faithful homologous recombination repair in S/G_2_ (10, 11). The ATR DNA-related pathway is activated in response to single-strand DNA breaks but can augment ATM activity and is mediated through the CHK1 kinase (12). In contrast, nonhomologous end joining uses different sets of factors, and repair occurs in G_1_ and is prone to error (13).

In high-risk HPV-positive cells, the ATM and ATR DNA repair pathways are constitutively activated in the absence of any external DNA damaging agents. DDR factors such as RAD51, CHK2, SMC1, FANCD2, H2AX, NBS1, RAD51, and MK2 are recruited to HPV genomes and are necessary for amplification (14–18). Short hairpin RNA-mediated knockdown or chemical inhibition of these factors leads to either impaired viral genome maintenance or differentiation-dependent amplification. This demonstrates an important role for the DNA damage repair pathways in the productive HPV life cycle. It is, however, still unclear how HPV activates the DDR and the mechanism by which repair factors contribute to viral replication.

In this study, we investigated whether double-strand breaks are induced in HPV-positive cells and if activation occurs as an indirect consequence of viral replication. Using both neutral comet assays and pulsed-field gel electrophoresis (PFGE), our studies demonstrated that expression of HPV proteins, in the absence of viral genome replication, is sufficient to cause double-strand breaks. Importantly, these breaks are found at high rates in chromosomal DNAs and minimally within the HPV genomes themselves. Furthermore, our studies indicate there is preferential recruitment of several DDR factors such as SMC1, RAD51, and BRCA1 to viral genomes, indicating that viral genomes are rapidly repaired in these cells, resulting in amplification.

## RESULTS

### HPV-positive cells contain high levels of double-strand DNA breaks.

**FIG 1:**
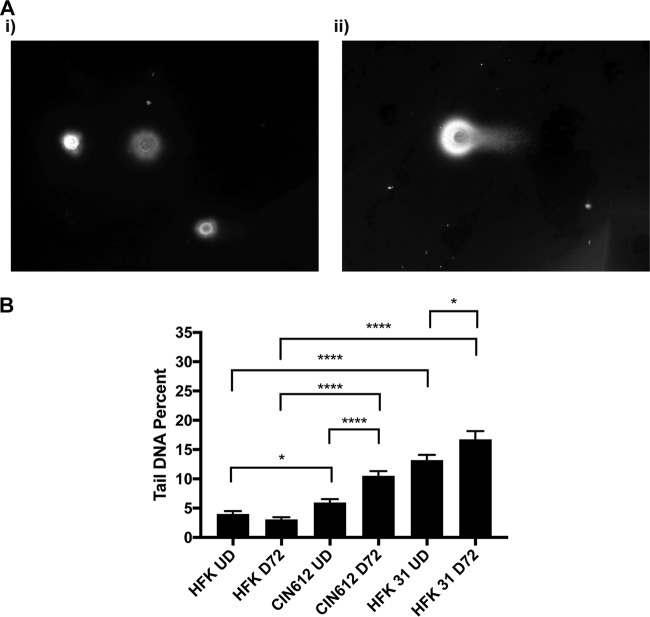
Episomal HPV-positive cells lead to DSB formation in a differentiation-dependent manner. (A) Neutral comet assays were performed on differentiated HFK (i) and CIN612 (ii) cells and visualized using a total DNA stain and epifluorescence microscopy. Panels are representative images of comets. (B) Neutral comet assays were performed on undifferentiated (UD) and 72-h calcium-induction-differentiated (D72) HFKs, CIN612 cells, and matched-genetic-background HFK 31 cells, which were visualized with epifluorescence microscopy and quantitated programmatically using the OpenComet software package for FIJI in percent tail DNA. Statistical significance is indicated on the graph and was analyzed using a Student’s *t* test and results from at least three individual experiments. ****, *P* < 0.0001; *, *P* < 0.05. Brackets indicate comparisons between conditions. More than 100 cells were analyzed for each condition across at least three individual experiments.

To investigate whether the constitutive activation of the ATM DNA damage pathway in high-risk HPV-positive cells is the result of virally induced double-strand breaks (DSBs), we first used the neutral comet assay, which involves electrophoresis of DNA from *in situ* lysed cells embedded in low-melting-temperature agarose and allows visualization of DSBs using a total DNA stain. In this assay, the nucleoid body contains intact genomic DNAs whereas small DNAs generated through DNA breaks are localized in the faster-migrating tail region. Comets are visualized using fluorescence microscopy, and the ratio of breaks to total DNA is quantitated using software that compares the signal in each comet tail to that in the corresponding nucleoid body. Controls using marker DNAs indicate that fragments smaller than 8 kb migrate into tails. Our studies showed that DSBs are present at a high level in HFK 31 and CIN612 cells in comparison to normal keratinocytes (Fig. 1Ai and ii). We also investigated how differentiation affects DSB formation and found that while DSBs increase in number in both sets of cells, there is a higher level in HPV-positive cells than in similarly differentiated human foreskin keratinocytes (HFKs) (Fig. 1B). These data indicate that DSBs are present in undifferentiated cells in cells that stably maintain HPV episomes and that the levels increase upon differentiation in a manner coincident with amplification.

We next examined the state of small DNAs in both normal keratinocytes and HPV-positive cells using PFGE. PFGE allows high-resolution separation of DNA species by size, and we used this method to compare breaks that were present in the total DNA of normal keratinocytes and in that of two high-risk HPV 31-positive cell lines. Total DNA was isolated from primary human foreskin keratinocytes (HFKs) along with two HPV 31-positive cell lines that stably maintain high-risk HPV 31 as episomes, HFK 31 and CIN612. HFK 31 was generated by transfection of HFKs with recircularized HPV 31 genomes, while CIN612 was derived from a patient biopsy specimen and stably maintains HPV 31 genomes as episomes. These cells were grown in monolayer cultures as well as following differentiation in high-calcium media. The HPV lifecycle is intimately linked to the differentiation program of stratified epithelia, as HPV episomes undergo amplification upon differentiation. This process can be accurately recapitulated using calcium-induced differentiation, which generates a uniformly differentiated population of cells. In keratinocytes with HPV episomes, amplification begins at 48 h after the calcium switch and plateaus at 72 to 96 h (2). DNA lysates were harvested from undifferentiated and differentiated cells and examined by Southern blot analysis following PFGE. To facilitate visualization of smaller DNA fragments, DNAs were first digested with XhoI, which lacks restriction sites in HPV 31, prior to electrophoresis. Fig. S1Ai in the supplemental material shows the presence of episomal copies of HPV 31 and that the levels increased upon differentiation as observed in previous studies (3, 19). No fragmentation of HPV supercoiled episomes was observed in undifferentiated or differentiated cells. The smear at the top of the gel in the HPV-positive lanes corresponds to the low-level integration of viral genomes seen in all analyses, which was retained at similar levels throughout passaging. To screen for total cellular sequences, a probe corresponding to the highly repetitive human Alu repeat sequence was generated and used in these analyses. The state of host DNAs is shown in Fig. S1Aii, with faster-migrating small DNAs detected in the total DNA from HPV-positive cells but not found in normal cells. In normal keratinocytes, smaller DNA fragments range in size from approximately 24 to 10 kb, with minimal signal at less than 10 kb. In contrast, small host DNA fragments less than 10 kB is size are present at high levels in HPV 31-positive cells. These results indicate that small DNA fragments are generated at a higher rate in HPV-positive cells than in normal keratinocytes due to double-strand break formation.

10.1128/mBio.00064-18.1 FIG S1  Pulsed-field electrophoresis demonstrates an accumulation of small DNAs in HPV-positive cells, and T4 DNA ligase assay demonstrates an increase in DSBs in differentiated HPV-positive cells. (A) Total DNA was extracted from undifferentiated (UD) and differentiated (D) normal HFKs, CIN612 cells, and matched-genetic-background HFK 31-positive cells, loaded into a gel, and run on a pulsed-field gel electrophoresis apparatus. Samples were examined by Southern blot analysis with a HPV 31 probe (i) and then stripped and probed with a human Alu repeat probe (ii). Data are representative of results from three experiments. (B) T4 DNA ligase assay in differentiated HFKs and CIN612 cells in the presence and absence of 5 µM KU55933 demonstrates that HPV-positive cells contain DSBs that accumulate in the presence of ATM inhibitor. The images presented are representative of results from three individual experiments. Cells were visualized with epifluorescence microscopy. Download FIG S1, TIF file, 3 MB. Copyright © 2018 Mehta and Laimins. 2018 Mehta and Laimins This content is distributed under the terms of the Creative Commons Attribution 4.0 International license.

Finally, similar findings were observed using a third assay, which utilizes T4 DNA ligase to identify the presence of DSBs. In this assay, T4 DNA ligase, ATP, and hairpin oligonucleotides containing a biotin tag were diffused into cells that had been lightly permeabilized with Triton X-100. These hairpin oligonucleotides can be ligated only to double-strand breaks and, after a wash to remove nonligated oligonucleotides, were observed by fluorescence microscopy. Only background signal was observed in undifferentiated normal keratinocytes and only a low level in undifferentiated HPV-positive cells. In contrast, consistently higher levels of positive signals were detected in differentiated HPV-positive cells (Fig. S1B). The data resulting from the use of multiple assays led us to conclude that double-strand breaks are formed in HPV-positive cells but not in normal keratinocytes and that the number of breaks increases upon differentiation.

### Expression of E6 and E7 lead to genetic instability in host DNAs.

**FIG 2:**
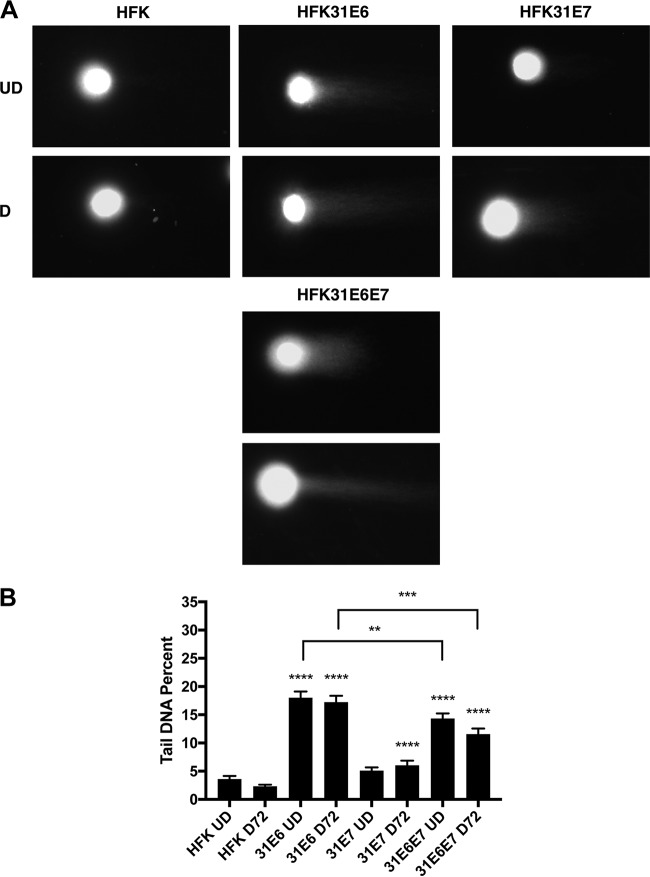
HPV oncogenes E6 and E7 induce DSBs by neutral comet assay. (A) Neutral comet assays were performed on undifferentiated or differentiated HFK, HFK 31E6, HFK 31E7, and HFK 31E6E7 cells and visualized using a total DNA stain and epifluorescence microscopy. Panels are representative images of comets. (B) Neutral comet assays were performed on undifferentiated and calcium-induction-differentiated (72-h) HFK, HFK 31E6, HFK 31E7, and HFK 31E6E7 cells, which were visualized with epifluorescence microscopy and quantitated programmatically using the OpenComet software package for FIJI in percent tail DNA. Statistical significance is indicated on the graph and was analyzed using a Student’s *t* test and results from at least three individual experiments. ****, *P* < 0.0001; ***, *P* < 0.001; **, *P* < 0.01. Statistical significance data indicate increases over the results seen with undifferentiated or differentiated normal keratinocytes. Brackets indicate statistical comparisons between E6 and E6/E7. More than 100 comets were analyzed for each condition across at least three individual experiments.

We next investigated whether expression of either of the two viral oncoproteins E6 and E7 alone was able to induce the formation of double-strand breaks or whether replication of viral genomes was required. Previous studies indicated that viral E6 and E7 expression from retroviral expression vectors is similar to that observed in episomal cell lines (20). Normal keratinocytes were transduced with retroviruses expressing HPV 31 E6 or HPV 31 E7 or the combination of HPV 31 E6 and E7 (HPV 31 E6E7), and stable lines were isolated. Following expansion, undifferentiated and calcium-differentiated cells were examined by neutral comet assays for the presence of DNA breaks. Interestingly, cells expressing E6 alone exhibited significant induction of DSBs independently of differentiation status compared to normal HFKs (Fig. 2). In contrast, undifferentiated cells expressing E7 alone exhibited only slightly elevated levels of DSBs in comparison to normal cells; however, upon differentiation, E7-expressing cells contained higher levels of breaks than HPV-negative cells (Fig. 2). Importantly, when E6 and E7 were coexpressed, DSB induction was increased to high levels that were comparable to the levels observed in lines with episomal forms of HPV 31 genomes as well as in cell lines solely expressing HPV E6 (Fig. 2B). This indicates that oncoprotein expression alone is sufficient for DSB induction and that replicating viral genomes are not required.

### Double-strand breaks are present primarily in cellular DNA and minimally in viral DNAs.

**FIG 3:**
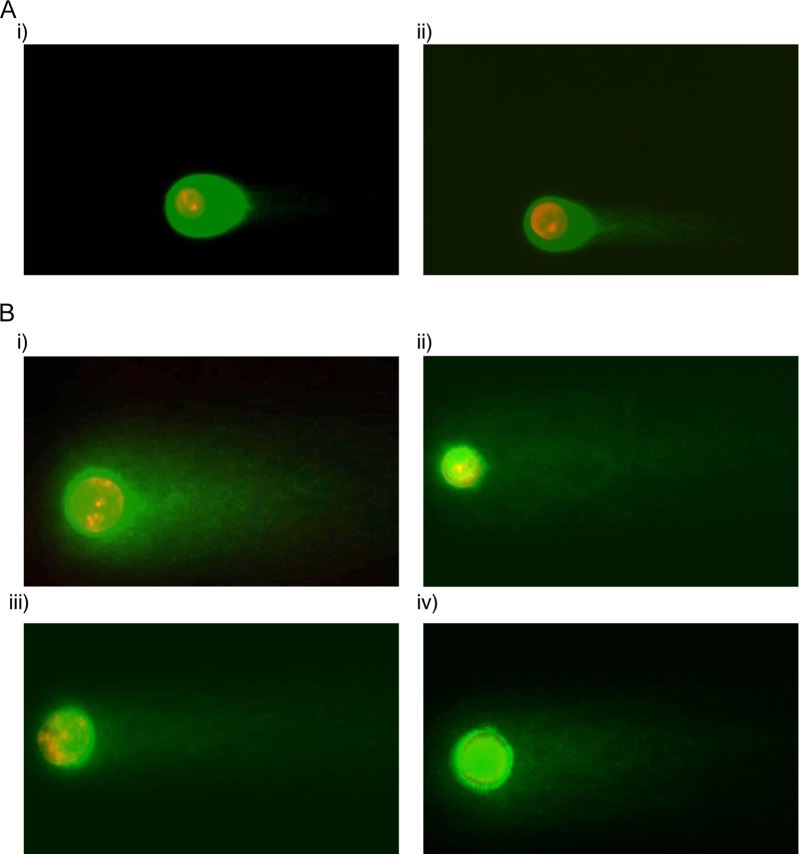
Double-strand breaks occur minimally within the HPV genome and are rapidly repaired by ATM or ATR. (A) DBD-FISH assays were performed on (i) undifferentiated or (ii) calcium-differentiated (72-h) HPV-positive cells, which were probed with an HPV 31 probe (red), stained for total DNA (green), and visualized using epifluorescence microscopy. (B) Undifferentiated HPV-positive cells were also treated with no drug (i), 10 μM ATM inhibitor (ATMi) KU-55933 (ii), 5 μM ATR inhibitor (ATRi) VE-822 (iii), or both ATM and ATR inhibitor (iv). Addition of both ATMi and ATRi led to loss of HPV signal in the nucleoid body. Data are representative of results from three individual experiments.

It was important to next determine whether these DSBs were being formed in cellular or viral DNAs. For these studies, a modified double-stranded break detection fluorescent *in situ* hybridization (DBD-FISH/Comet-FISH) assay was used that combines the neutral comet assay with FISH for HPV genomes. Total cellular DNA from HFK 31 cells was first subjected to a comet assay, after which separated DNAs were hybridized with HPV 31 probes and the signal was amplified, incorporating a red nucleotide fluorophore. Total DNA was visualized with a DAPI (4′,6-diamidino-2-phenylindole) stain (in green). Fluorescence microscopy examination of the results from these DBD-FISH assays identified a defined region of HPV DNA in the nucleoid body with little if any signal in the tail in undifferentiated cells (Fig. 3Ai). Upon 72 h of calcium-induced differentiation, the HPV signal increased in intensity and remained localized to the nucleoid body with little to no signal in the tail (Fig. 3Aii). No red signal indicative of HPV DNAs was observed in normal HFK controls (Fig. S2i and ii). Pretreatment of cells with either the ATM inhibitor KU-55933 or the ATR inhibitor VE-822 prior analysis had only a modest effect on the migration of the HPV DNA signal from the nucleoid body into the tail or resulted in only a modest reduction in levels (Fig. 3Bii and iii). In contrast, when cells were treated with both ATM and ATR inhibitors (Fig. 3Biv), no signal for HPV DNA was detectable in the nucleoid body, indicating that it had migrated into the tail. This demonstrates that the DSBs induced in HPV-positive cells are present at high levels in host chromosomal DNAs and minimally in the HPV genome. Furthermore, the results of this analysis indicate that both ATM and ATR are important for maintaining the integrity of viral genomes, likely by allowing rapid repair of HPV DNA. This is consistent with observations showing that both ATM activation and ATR activation are required for genome amplification.

10.1128/mBio.00064-18.2 FIG S2  Normal keratinocytes do not have HPV FISH signal. DBD-FISH assays were performed on normal HFKs that were (i) undifferentiated or (ii) calcium differentiated (72 h) and probed with an HPV 31 probe (red), stained for total DNA (green), and visualized using epifluorescence microscopy. Download FIG S2, TIF file, 0.6 MB. Copyright © 2018 Mehta and Laimins. 2018 Mehta and Laimins This content is distributed under the terms of the Creative Commons Attribution 4.0 International license.

### Double-strand breaks and newly replicated DNAs.

**FIG 4:**
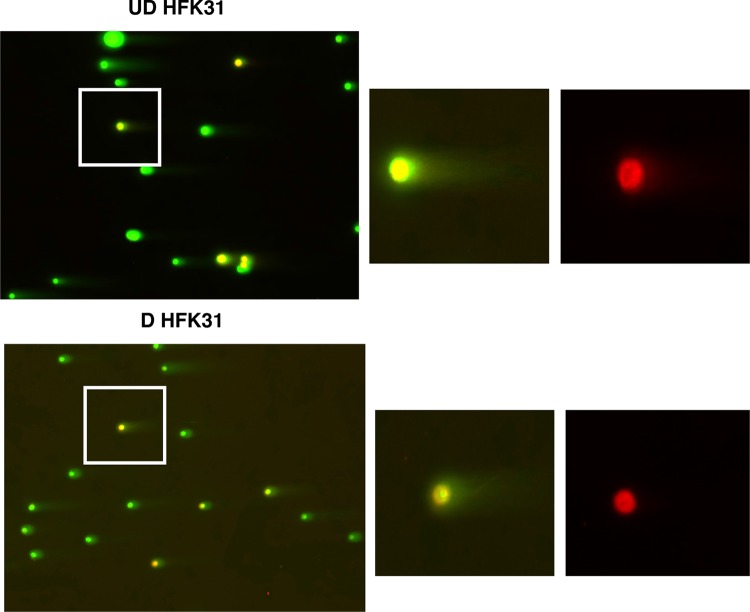
Comet EdU assays demonstrate that nascent DNA contains minimal breaks upon calcium-induced differentiation. Comet-EdU assays were performed on undifferentiated and calcium-differentiated HPV-positive cells, which were visualized using epifluorescence microscopy. Cells were pulse labeled with EdU followed by neutral comet assays and stained for total DNA and EdU. Green signal indicates total DNA stain, while red signal indicates nascent DNA (EdU). Undifferentiated cells contain a positive signal in the red tail, indicating the presence of breaks in nascent DNA, while differentiated cells contain minimal red signal in the tail, indicating minimal breaks in nascent DNA upon differentiation. Images are representative images from three individual experiments.

To examine whether DSBs are present in newly replicated DNAs, we developed a novel assay that combines the neutral comet assay with EdU labeling. This technique identifies newly replicated DNAs and allows investigation of the *de novo* induction of DSBs in HPV-positive cells. In this assay, total DNA is identified with a green stain whereas red staining corresponds to newly replicated DNA. Labeling undifferentiated HPV-positive cells with EdU showed an overlap of red and green signals in both the nucleoid body and the tail of the comet consistent with the idea that DNA breaks are present in the cellular DNAs that constitute the majority of newly replicated DNAs (Fig. 4). At 72 h of differentiation, newly replicated DNA was restricted to the nucleoid body and was minimally present in comet tails. This indicates that few, if any, DNA breaks are retained in amplifying HPV genomes upon differentiation. Staining for total DNA shows a tail length consistent with the generation of breaks during replication of cellular DNA during S phase. On the basis of the DBD-FISH results and findings from our neutral comet assays, we conclude that DSBs are present at a high level within the host DNA but at a low level in HPV genomes and that this occurs in a differentiation-dependent manner.

### DNA repair factors are preferentially recruited to viral genomes.

**FIG 5:**
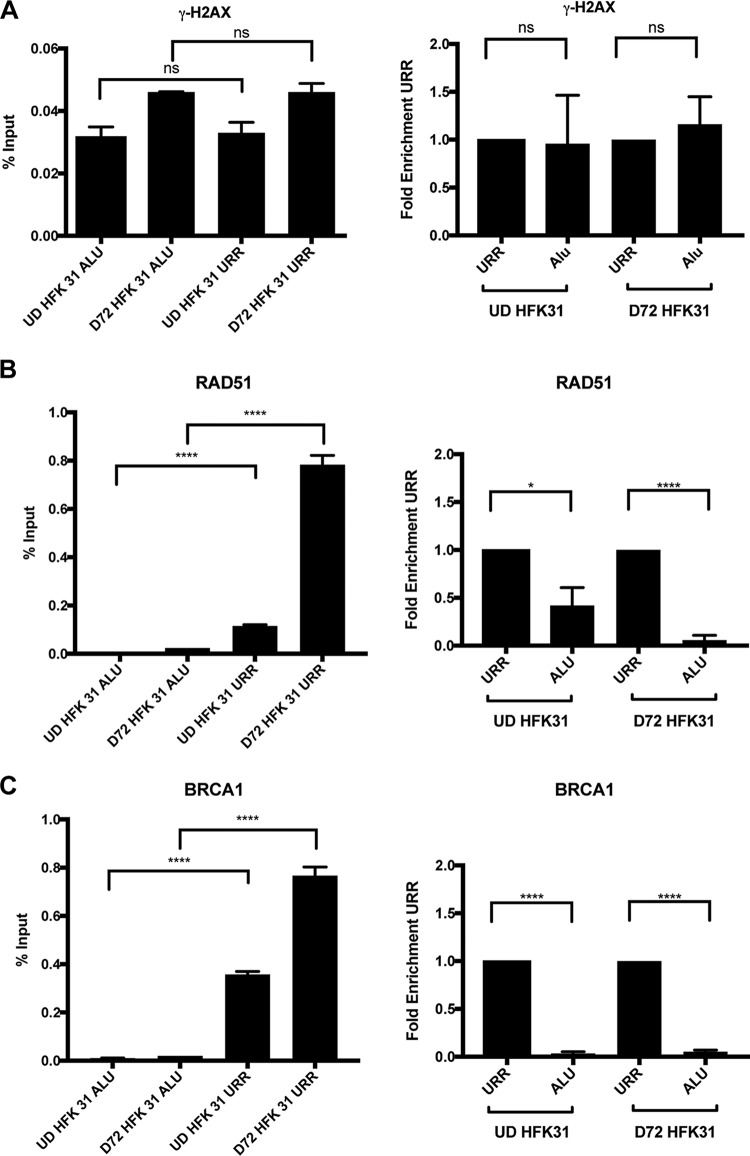
DNA repair factors are preferentially recruited to viral genomes. Chromatin immunoprecipitation analysis was performed on undifferentiated and differentiated HPV-positive cells. (A) ɣ-H2AX, (B) RAD51, and (C) BRCA1 bind at higher levels to the URR of HPV 31 than to the human Alu repeat, and the difference increases upon calcium-induced differentiation. Data are represented as percent input or normalized to the URR as fold enrichment over URR per differentiation condition. For fold enrichment measurements, a value of less than one indicates less binding to Alu than the URR. Similar results were observed in at least three independent experiments. Statistical significance is indicated on the graph and was analyzed using a Student’s *t* test. ****, *P* < 0.0001; *, *P* < 0.05; ns, not significant. Statistical significance data indicate results of comparisons between Alu occupancy and URR occupancy for either undifferentiated or differentiated HPV-positive cells. All primer efficiencies were determined to be comparable. IgG background signal data are shown in the supplement material.

The studies described above indicate that DNA breaks are preferentially present in host chromosomal DNAs in comparison to viral genomes. One explanation for this observation is that breaks are induced at the same rates in viral and cellular sequences but repaired at higher rates in viral genomes, which is consistent with our observations by comet-FISH using the combination of ATM and ATR inhibitors. Such a model would suggest that DNA repair factors are preferentially recruited to viral genomes for rapid repair. To test this hypothesis, we used chromatin immunoprecipitation (ChIP) analyses to investigate DDR factor occupancy of host and viral genomes. Using matched normal keratinocytes and HPV 31-positive keratinocytes, the ChIP assays were first performed for the DSB marker ɣ-H2AX by assessing its occupancy in both the HPV upstream regulatory region (URR), which was previously shown to be occupied with active H2AX, and the cellular DNAs represented by Alu repeats (14). Our studies indicate that ɣ-H2AX is recruited to HPV DNAs in undifferentiated cells at levels similar to those seen with cellular DNAs. SMC1 is also recruited to HPV DNA at levels similar to those seen with the host (Fig. 5A; see also Fig. S3A). Upon differentiation, the amount of binding to cellular DNAs increased but the level was substantially higher with the viral genomes. Next, we examined the binding of DNA damage factors RAD51 and BRCA1 to viral and cellular DNA. Interestingly, we found substantially increased binding of these factors to viral DNAs compared to the host DNA. The binding of RAD51 to viral DNA was particularly enhanced (by 5-fold to 10-fold) upon differentiation compared to the results seen with cellular DNAs (Fig. 5B and C). We conclude that there is preferential recruitment of a subset of DNA damage repair factors to viral genomes compared to host chromosomal sequences.

10.1128/mBio.00064-18.3 FIG S3  SMC1 binds to the host genome and the HPV genome at similar levels, and IgG background signal was examined in chromatin immunoprecipitation analyses. (A) Chromatin immunoprecipitation analysis was performed on undifferentiated and differentiated HPV-positive cells in the presence and absence of 3 mM hydroxyurea. SMC1 binds to the URR of HPV 31 at levels similar to those seen with human Alu in the presence or absence of hydroxyurea. Data are normalized to the URR as fold enrichment over URR per differentiation condition. Similar results were observed in at least three independent experiments. Statistical significance is indicated on the graph and was analyzed using a Student’s *t* test. Statistical significance data indicate results of comparisons between Alu occupancy and URR occupancy for either undifferentiated or differentiated HPV-positive cells. All primer efficiencies were determined to be comparable. (B) Chromatin immunoprecipitation analysis of IgG background signal for ɣ-H2AX was performed on undifferentiated HPV-positive cells in the presence of increasing concentrations of hydroxyurea (3 mM, 5 mM, and 7.5 mM) for 72 h. The data in the graph represent the IgG background signal observed. (A) Undifferentiated Alu. (B) Undifferentiated Alu plus 3 mM HU. (C) Undifferentiated Alu plus 5 mM HU. (D) Undifferentiated Alu plus 7.5 mM HU. (E) Undifferentiated URR. (F) Undifferentiated URR plus 3 mM HU. (G) Undifferentiated URR plus 5 mM HU. (H) Undifferentiated URR plus 7.5 mM HU. Data are representative of results from three individual experiments. (C) Chromatin immunoprecipitation analysis of IgG background signal for RAD51 and BRCA1 was performed on undifferentiated and differentiated HPV-positive cells treated with or without HU for 72 h. Little signal is observed across all samples. (A) Undifferentiated Alu. (B) 72-h-differentiated Alu. (C) Undifferentiated Alu plus 3 mM HU. (D) 72-h-differentiated Alu plus 3 mM HU. (E) Undifferentiated URR. (F) 72-h-differentiated URR. (G) Undifferentiated URR plus 3 mM HU. (H) 72-h-differentiated URR plus 3 mM HU. Data are representative of results from three experiments. Download FIG S3, TIF file, 0.4 MB. Copyright © 2018 Mehta and Laimins. 2018 Mehta and Laimins This content is distributed under the terms of the Creative Commons Attribution 4.0 International license.

RAD51 and BRCA1 play important roles in the repair of DNA breaks induced following replication fork collapse. One way that fork collapse can occur is as a result of collisions between replication machinery and transcriptional machinery as observed in other episomal systems (21). The DNA/RNA hybrid R-loops that form during these collisions generate double-strand breaks and require DDR pathways for resolution (22). BRCA1 is important for resolution of R-loop structures that lead to fork stalls, and RAD51 is important for stalled replication fork restart following exposure to genotoxic stresses such as those that occur in the presence of R-loops (23, 24). We therefore investigated if R-loops form on HPV genomes through the use of a DNA/RNA hybrid-specific antibody in DNA/RNA immunoprecipitation (DRiP) assays. Our studies suggested that R-loops form on HPV genomes in undifferentiated cells but are lost or rapidly resolved upon differentiation (Fig. S4).

10.1128/mBio.00064-18.4 FIG S4  The HPV 31 genome contains R-loops that reduce in number upon differentiation. DNA-RNA hybrid immunoprecipitation analysis was performed on undifferentiated and differentiated HPV-positive cells. R-loops are present at the HPV 31 URR and decrease in number upon calcium-induced differentiation. Download FIG S4, TIF file, 0.1 MB. Copyright © 2018 Mehta and Laimins. 2018 Mehta and Laimins This content is distributed under the terms of the Creative Commons Attribution 4.0 International license.

### Effect of hydroxyurea on viral and cellular DNAs.

**FIG 6:**
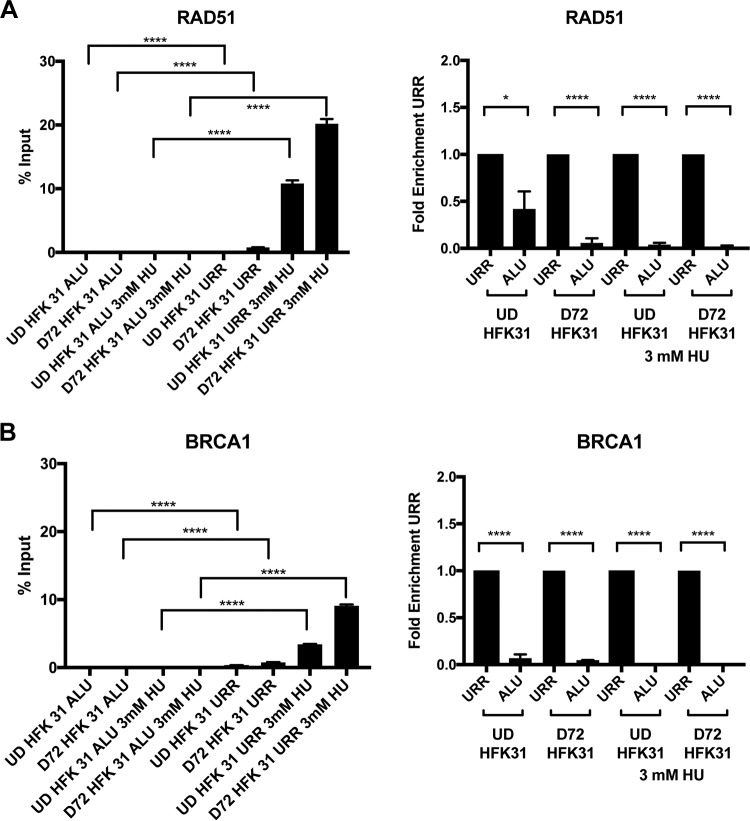
HU treatment results in recruitment of HR factors to viral DNA. Chromatin immunoprecipitation analysis was performed on undifferentiated and differentiated HPV-positive cells in the presence and absence of 3 mM hydroxyurea. (A) RAD51 and (B) BRCA1 bind to the URR of HPV 31 at higher levels than to the human Alu in the presence of hydroxyurea. Data are represented as percent input (left column) or (normalized to the URR) as fold enrichment over URR (right column) per differentiation condition. For fold enrichment measurements, a value of less than 1 indicates less binding to Alu than to the URR. Similar results were observed in at least three independent experiments. Statistical significance is indicated on the graph and was analyzed using a Student’s *t* test. ****, *P* < 0.0001; *, *P* < 0.05. Statistical significance data indicate results of comparisons between Alu occupancy and URR occupancy for either undifferentiated or differentiated HPV-positive cells. All primer efficiencies were determined to be comparable.

Given the preferential recruitment of RAD51 and BRCA1 to viral genes, we next investigated the effect of adding the DNA damaging agent hydroxyurea (HU), which specifically induces replication fork collapse by depleting nucleotide pools, to cells that stably maintain HPV episomes. We first performed a titration with increasing amounts of hydroxyurea on cells with viral episomes and screened for binding of ɣ-H2AX to both host Alu repeats and the upstream regulatory region (URR) of HPV 31. We determined that increasing the concentrations of HU resulted in increased recruitment of ɣ-H2AX to both host DNA and viral DNA. Interestingly, the binding of ɣ-H2AX to HPV DNA was moderately lower than to cellular DNA, suggesting a differential DNA damage response for viral DNA (Fig. S5). It was next important to determine if HU treatment altered the levels of binding of other DNA damage response factors to viral genomes and whether this changed with differentiation. For this analysis, we used 3 mM HU as a representative concentration and found that BRCA1 and RAD51 were preferentially recruited to the HPV genome during genotoxic stress at very high levels, occupying nearly approximately 10% to 20% of the HPV genomes, while binding to the human genome remained at very low levels (Fig. 6). SMC1, however, was recruited similarly to the host DNA and viral DNA in the presence of HU (Fig. S3A). This analysis suggests that RAD51 and BRCA1 are recruited at very high levels under conditions of genotoxic stress resulting from replication fork collapse.

10.1128/mBio.00064-18.5 FIG S5  HU induces ɣ-H2AX recuitment to both host and viral DNA. Chromatin immunoprecipitation analysis was performed on undifferentiated CIN612 cells in the presence of increasing concentrations of hydroxyurea (3 mM, 5 mM, and 7.5 mM) for 72 h. ɣ-H2AX binds at higher levels to the human Alu repeat sequences with increases in HU concentration. Binding of ɣ-H2AX to HPV URR increases with HU concentration but to a lesser level than to host DNA. Download FIG S5, TIF file, 0.2 MB. Copyright © 2018 Mehta and Laimins. 2018 Mehta and Laimins This content is distributed under the terms of the Creative Commons Attribution 4.0 International license.

### HPV-positive cells maintain HR factor expression in the presence of HU.

**FIG 7:**
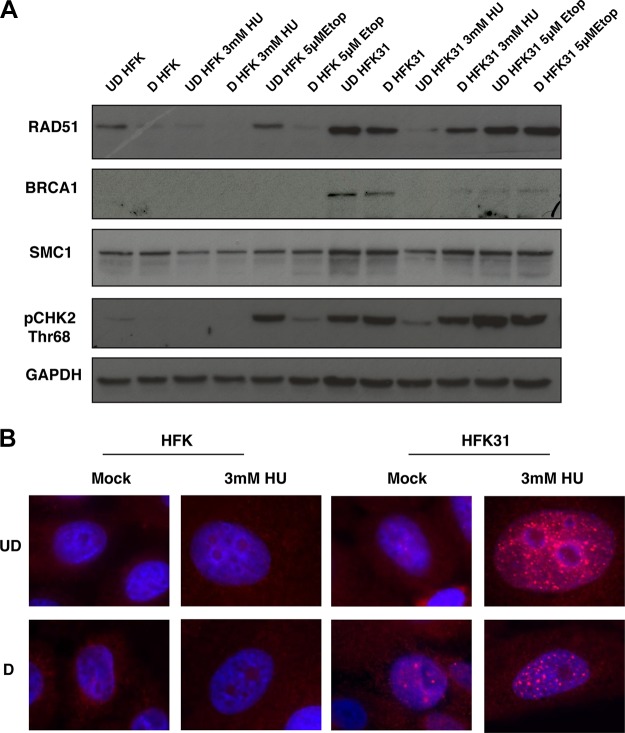
DDR factors are upregulated in HPV-positive cells and maintained in the presence of HU, and RAD51 foci are maintained in HPV-positive cells with HU treatment. (A) Whole-cell protein lysates were isolated from HFKs as well as from matched-genetic-background HFK 31 cells and analyzed by Western blotting with antibodies against RAD51, BRCA1, SMC1, pCHK2 threonine 68, and GAPDH as a loading control. Western blot data are representative of results from three individual experiments. (B) Immunofluorescence for RAD51 in undifferentiated and differentiated HFKs and matched-background HFK 31 cells in the presence and absence of 3 mM HU demonstrates that HPV-positive cells maintain RAD51 foci in the presence in HU, whereas normal HFKs lack these foci in both treated and untreated cells. The images presented are representative of results from three individual experiments.

The ChIP analysis described above indicated that large portions of HPV genomes were occupied by RAD51 and BRCA1 in the presence of HU. Previous reports found that HPV increases the expression of many components of the DDR, and several of these proteins, including RAD51 and BRCA1, were shown to colocalize with the HPV genome using immunofluorescence-FISH. To test this, we first examined the levels of various members of the DDR in the presence and absence of genotoxic stress agents using Western blot analysis. Interestingly, although RAD51 and BRCA1 levels were reduced in HFKs in the presence of HU, HPV-positive cells maintained higher levels of BRCA1 and RAD51, as well as of pCHK2 (Fig. 7A; see also Fig. S6). Similar increases in the number of RAD51 foci were seen following treatment with 3 mM HU as well (Fig. 7B). For comparison, we examined the effect of treatment with another DNA damaging agent, the topoisomerase II inhibitor etoposide, which does not induce replication fork collapse, and observed no change in RAD51 levels (Fig. 7A).

10.1128/mBio.00064-18.6 FIG S6  Western blot densitometry analysis. Data represent averages of results from densitometry analysis performed using FIJI across three individual Western blot experiments whose results are shown in Fig. 7. Download FIG S6, TIF file, 0.3 MB. Copyright © 2018 Mehta and Laimins. 2018 Mehta and Laimins This content is distributed under the terms of the Creative Commons Attribution 4.0 International license.

**FIG 8:**
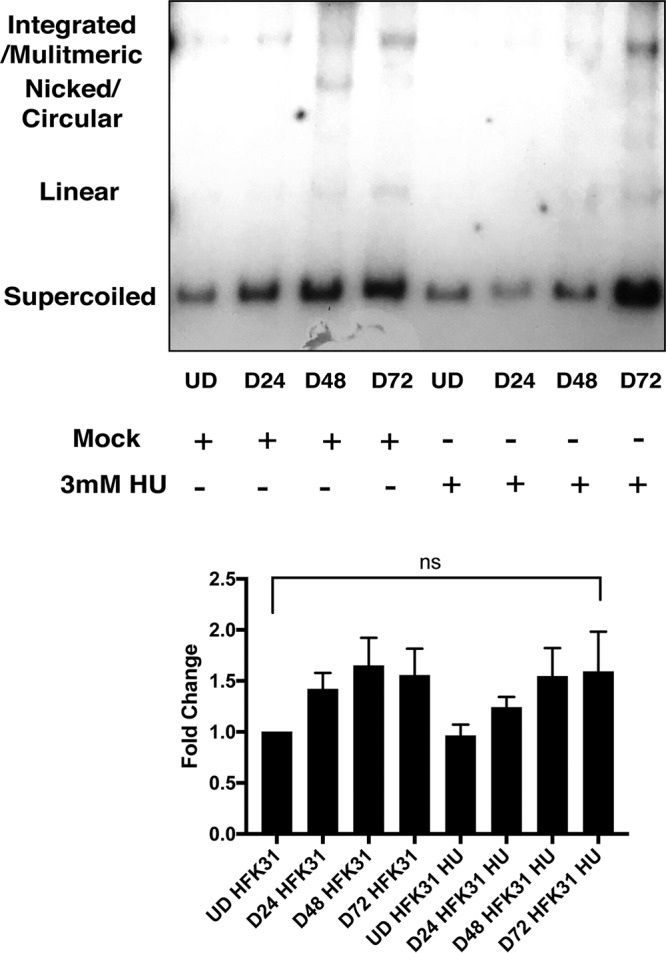
HPV differentiation-dependent amplification occurs normally in the presence of HU. HFK 31 cells were induced to differentiate by the addition of calcium for 24, 48, and 72 h in the presence and absence of 3 mM HU and analyzed by Southern blotting. No change was seen in the ability of HPV to amplify. Densitometry analysis was performed using FIJI across three individual experiments, and data are normalized to undifferentiated HFK 31 cells. Statistical significance is indicated on the graph and was analyzed using a Student’s *t* test. No significant change was observed in the presence of hydroxyurea.

Finally, to assess whether HU treatment affected HPV amplification, we examined DNAs isolated from HPV-positive cells in the presence and absence of HU by Southern blot analysis. While cellular DNA replication was inhibited by HU treatment, amplification of HPV genomes upon differentiation still occurred at comparable levels, suggesting that HPV can recruit and use DDR factors for its viral replication even when host DNA is severely damaged (Fig. 8).

## DISCUSSION

High-risk human papillomaviruses activate the ATM and ATR DNA damage repair pathways in the absence of external DNA damaging agents to mediate viral genome amplification. Our studies showed that activation of these pathways occurs as a result of double-stranded DNA breaks induced by HPV proteins and independently of viral replication. In cells that stably maintain viral episomes, low levels of breaks are present in undifferentiated cells and the levels increase substantially upon differentiation concurrently with genome amplification. Importantly, our studies showed that DNA breaks are primarily found in cellular DNAs and minimally in viral genomes. This could mean either that the breaks are preferentially induced only in cellular loci or that breaks are induced in both viral and cellular DNAs but are rapidly repaired specifically in HPV genomes. Our findings provide support for the latter conjecture, as treating cells that maintain HPV genomes with inhibitors for both ATM and ATR leads to formation of DNA breaks and fragmentation of viral episomes. This indicates that ATM and ATR act cooperatively to repair HPV genomes and is consistent with studies showing that activation of both ATM and ATR is critical for HPV genome amplification. Furthermore, ATR has previously been shown to complement the activity of ATM (25), which is also consistent with our observations. The use of EdU labeling to identify newly replicated DNAs in conjunction with comet assays provides additional support for the idea that minimal breaks are present in replicating HPV genomes. In undifferentiated cells, a high level of cellular replication occurs along with low levels of HPV maintenance replication. Since replication of cellular DNA far exceeds that of viral genomes, the majority of the EdU signal in undifferentiated cells is incorporated into cellular DNA, which contains many breaks. Upon the calcium-induced differentiation of cells with HPV episomes, cellular replication occurs in S phase prior to HPV genome amplification in G_2_. Importantly, viral amplification occurs only after cellular replication has finished (26, 27). When we examined EdU labeling during late differentiation, the majority of signal was incorporated into newly replicated viral genomes, which had minimal levels of breaks. Overall, these observations indicate that DNA breaks are induced in both cellular and viral DNAs but that repair of viral genomes occurs rapidly.

The preferential repair of the DNA breaks in viral genomes is likely a result of repair factors being recruited at higher levels to HPV episomes than to cellular DNAs. Previous studies indicated that Alu repetitive elements are prone to homologous recombination, replication fork stalling, and genetic rearrangements (28). Our studies showed that some DDR factors such as SMC1 are recruited at similar levels to HPV genomes and to cellular DNAs (see Fig. S3A in the supplemental material). In contrast, RAD51 and BRCA1 are recruited to viral DNAs at substantially higher levels than to cellular sequences and the difference is further increased upon differentiation. Both RAD51 and BRCA1 mediate homologous recombination repair in response to collapsed replication forks, suggesting that this may be the mechanism by which breaks are generated in viral genomes. When HPV-positive cells are treated with hydroxyurea, a drug that specifically induces replication fork collapse, BRCA1 and RAD51 were recruited to viral genomes at even higher levels, with minimal binding at cellular loci. Furthermore, treatment of normal cells with HU rapidly reduced the total levels of RAD51; however, higher levels were retained in similarly treated HPV-positive cells. Treating cells with hydroxyurea induced high levels of breaks in cellular DNAs, but this did not affect HPV genome amplification. The results of the studies described above suggest that factors critical for repair of collapsed replication fork-induced DNA damage are recruited at high levels to HPV genomes.

Previous studies showed that the E6 and E7 proteins can activate DNA damage repair pathways, but it was not clear if this was the result of break formation or if activation occurred by another mechanism such as direct binding to DDR proteins (29). Using comet assays, the results of our studies showed that expression of either E6 or E7 is sufficient to induce DNA breaks in cells and are consistent with a previous report by Duensing and Münger (30). While E6 induces high levels of breaks in both undifferentiated and differentiated cells, E7 acts primarily upon differentiation to induce breaks. The combination of the two oncoproteins is most effective and induces levels of breaks comparable to those seen in cells that stably maintain viral episomes. Interestingly, viral replication or amplification did not increase the levels of DNA breaks beyond those seen by expressing the oncoproteins by themselves. Previous work has shown that the levels of E6/E7 expression from retroviral vectors are similar to those seen with viral genomes (20). The E7 protein has been reported to activate DDR factors through its effects on E2F proteins; however, the mechanism by which E6 acts has not been demonstrated and may be related to its effects on p53. In our studies, we have used multiple methods to confirm the presence of DNA breaks in undifferentiated cells as well following differentiation.

**FIG 9:**
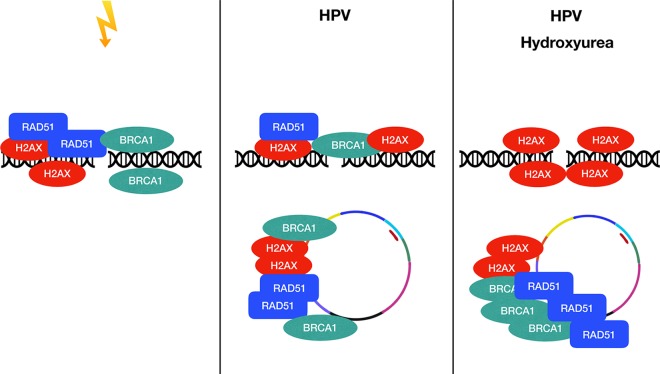
HPV preferentially and differentially recruits DDR factors to its own genome for productive replication. Under conditions of genotoxic stress, DDR factors are amplified on host DNAs. In the presence of HPV or viral E6 and E7, DDR factors are preferentially recruited to the HPV DNA, leading to DNA lesions and genetic instability in the host genome. This recruitment is amplified in the presence of hydroxyurea treatment.

The findings described above indicate that high-risk HPVs amplify their genomes in a differentiation-dependent manner by preferentially recruiting DDR factors away from breaks in the host DNA, leading to rapid repair of episomes and amplification. In this model, DDR factors in HPV-negative cells are recruited to genomic DNA to maintain genetic fidelity during stress (Fig. 9). In HPV-positive cells, DDR factors are recruited to viral genomes for productive replication and away from the host DNA. In the presence of genotoxic stress induced by hydroxyurea, DDR factors are preferentially recruited to viral genomes that would normally be bound to host DNA at high levels, and this allows productive viral replication in cells that do not normally divide. An issue still remains as to how HPV proteins induce DNA break formation. One possibility is that replication fork collapse is responsible for the induction of DNA breaks. Replication fork collapse can occur as a result of decreased levels of nucleotide pools induced by an increase in the number of origins firing in HPV-positive cells as a result of E7’s effects on retinoblastomas (Rb) and deregulation of E2Fs as suggested in a previous study (31). The effects of decreased levels of nucleotide pools mediated by viral oncogenes can potentially be overcome by activation of c-myc expression or through addition of exogenous nucleotides. Increased replication fork progression rates have been observed in HPV-positive cells, and such increases may also lead to collisions between replication machinery and transcriptional machinery as observed in other episomal systems (21). The RNA/DNA hybrid R-loops that form during these collisions generate double-strand breaks and require DDR pathways for resolution. Our initial studies suggested that HPV-positive cells contain high levels of R-loops and that R-loops on HPV genomes are lost or rapidly repaired upon differentiation and could be regulated by E6 and E7. The presence of R-loops in HPV-positive cells has been previously observed using electron microscopy (32). The induction of DNA breaks in HPV-positive cells could also occur through the action of the cytidine deaminase APOBEC3, whose levels are increased in HPV-positive cells (33). It is also possible that all these mechanisms are interconnected, and this issue needs to be addressed in future studies. It is also still unclear how DDR factors are preferentially recruited to HPV genomes. The E2 and E1 DNA binding proteins are good candidates as recruitment factors that interact with cellular proteins to bring them to viral genomes. For instance, E1 recruits cellular replication enzymes to viral origins in undifferentiated cells and it is possible that it also recruits DNA damage factors (34). Understanding how DDR factors are recruited to HPV genomes will require a detailed examination of the factors present at replication forks during amplification and in the basal state. Overall, these studies have demonstrated that HPV proteins induce breaks in host and viral DNA and that HPV preferentially recruits DDR components to its own genome for rapid repair and productive replication.

## MATERIALS AND METHODS

### Isolation of human foreskin keratinocytes.

Human foreskin keratinocytes (HFKs) were isolated from neonatal human epidermis. Deidentified neonatal foreskins submerged in sterile Hanks’ balanced salt solution (HBSS) transport media were obtained from the Skin Disease and Research Core at Northwestern University, and the isolations were performed within 72 h of circumcision. The foreskin was rinsed three times with normal phosphate-buffered saline (PBS) to remove transport media and transferred to a 100-mm-diameter dish along with 5 ml of PBS. The foreskin was cut open and laid flat using a sterile dissecting scissors and fine forceps and placed epidermis side down in the dish. Excess blood vessels, tissue, and fat were removed from the foreskin and discarded. The foreskin was cut into smaller pieces and transferred to a 60-mm-diameter dish epidermis side up and incubated overnight at 4°C in 4 ml of 2.4 U/ml Dispase (Roche). After 16 to 18 h of incubation, the epidermis was removed from the dermis using forceps and transferred to a new 100-mm-diameter dish containing 4 ml of prewarmed 0.25% trypsin (Invtrogen) and subsequently incubated at 37°C for 10 to 15 min. Following incubation, the epidermis was scraped against the bottom of the plate 20 to 30 times to dissociate the keratinocytes, and the trypsin was quenched using 500 µl of bovine serum (Life Tech). The dissociated cell suspension were transferred to a 50-ml conical tube and pipetted through a 40-µm-pore-size cell sieve, and the plate was rinsed with 5 ml of PBS and added to the 50-ml conical tube. The filtered cell suspension was transferred to a 15-ml conical tube and centrifuged for 5 min at 100 rpm. The supernatant was removed, and the cells were suspended in M154 (Life Tech) media supplemented with 0.07 mM calcium chloride. The medium was replaced the next day and then as required.

### Cell culture.

Primary HFKs were cultured in either M154 supplemented with 0.07 mM CaCl_2_ or E-medium supplemented with 5 ng/ml mouse epidermal growth factor (EGF) (Becton Dickinson, Franklin Lakes, NJ; catalog no. 354010). E-medium is Dulbecco’s modified Eagle's medium (DMEM) supplemented with multiple factors essential for keratinocyte growth, including Ham’s F-12 (Life Tech), NaHCO_3_ (Life Tech), penicillin-streptomycin (Pen-Strep; Life Tech), hydrocortisone (Sigma), cholera enterotoxin (Sigma), defined fetal bovine serum (FBS) (Sigma), adenine (Sigma), insulin (Sigma), transferrin, 3,3,5-triiodo-l-thyronine (T3; Sigma), and HEPES (Sigma) (35). All cells cultured in E-medium were cocultured on NIH 3T3-J2 fibroblasts arrested with 100 μl in 5 ml of mitomycin-c media (MEDAC) (0.4 mg/ml in PBS) for 1.5 h. CIN612 cells, which maintain the HPV 31 viral genome episomally, were cultured in E-medium plus EGF, with mitomycin-c-treated J2 feeders as well. J2 feeders were removed by incubating cells with Versene (PBS plus 0.05 mM EDTA) for 5 min and then rinsing the cells with PBS 2 to 3 times. 293T cells and PT67 Retropack J2 feeders were cultured in DMEM plus 1% penicillin-streptomycin (Life Tech) (5,000 U/ml) and 10% bovine serum (Life Tech). All cell lines were routinely tested for mycoplasma contamination.

### Plasmids.

pBR-322min containing HPV 31 was used for generating stable HPV 31 cell lines (HFK 31), and a pSV-Neo2 neomycin resistance plasmid was used for stable cell line selection. HPV oncogene stable cell lines were made using pLXSN, pLXSN 31E6, pLXSN 31E7, and pLXSN31E6E7 retroviral plasmids.

### Generation of cell lines that stably maintain HPV 31 episomes.

Recircularized HPV 31 was created by digesting the pBR-322 backbone from pBR-322min–HPV31 and subsequent religation. One microgram of recircularized HPV 31 DNA was contransfected with 1 μg of neomycin resistance expression vector (PSV2neo) using FuGene-6 (Roche) into one million freshly isolated HFKs in a 60-mm-diameter dish at 60% confluence. After 24 h, cells were transferred to a 100-mm-diameter dish. Cells were selected 24 h later with 200 μg/ml G418 followed by fresh mitomycin-treated J2 feeders the next day. J2 treatment was followed by one more course of 200 μg/ml G418 treatment and by replenishment of J2 feeders a day after. The same schedule was followed subsequently, alternating between treatment with 100 μg/ml G418 per day and feeding with J2 feeders. Cells were then expanded as previously described (36).

### Retroviral expression of HPV oncogenes.

Retroviral constructs (empty, HPV 31 E6 or E7, or HPV 31 E6E7) (3 μg) were transfected into 50% confluence PT67 Retropack cells using X-tremeGENE HP DNA transfection reagent (Roche, Indianapolis, IN; catalog no. 06366236001). The medium was changed 24 h posttransfection, and the cells were allowed to grow for another 24 h. The viral supernatants were collected and concentrated using a Millipore concentrator (catalog no. UFC910096). Two million freshly isolated HFKs were seeded and allowed to grow for 24 h. The concentrated viral particles were added to cells in the presence of 0.8 μg/ml Polybrene (Sigma-Aldrich)–3 ml of media to decrease the total volume for infection. After 6 h, an additional 4 ml of media was added. The medium was changed 24 h posttransduction, and the cells were allowed to grow for another 24 h. Cells were then selected using the same selection strategy as that outlined for the generation of stable HPV 31 HFK lines.

### Calcium-induced differentiation.

HFKs, CIN612 cells, and HFK 31 cells were grown to 60% to 70% confluence and subsequently switched to M154 (Life Tech) with human keratinocyte growth supplement (HKGS), 1% Pen-Strep, and 0.03 mM CaCl_2_ for 24 h. The next day, the medium was changed to M154 without HKGS, 1% Pen-Strep, and 1.5 mM CaCl_2_, and the reaction mixture was cultured for 72 h to induce differentiation.

### Western blot analysis.

Whole-cell lysates were extracted using radioimmunoprecipitation assay (RIPA) lysis buffer (50 mM Tris [pH 7.4], 150 mM NaCl, 0.1% Triton X-100, 0.25% sodium deoxycholate, 1% NP-40, 1 mM EDTA, pH 8.5) enriched with sodium pyrophosphate (2.5 mM), sodium fluoride (1 mM), sodium vanadate (1 mM), protease inhibitor cocktail (Roche), and phenylmethylsulfonyl fluoride (PMSF) (1 mM), following removal of NIH 3T3-J2 fibroblasts. Western blotting was performed as previously described (16).

### Antibodies.

The antibodies used were phospho-CHK2-Thr-68 (Cell Signaling; catalog no. 2661), SMC1 (Abcam, Inc.; catalog no. ab9262), GAPDH (glyceraldehyde-3-phosphate dehydrogenase; Santa Cruz), RAD51 (Millipore; catalog no. 05-530-I), BRCA1 (Thermo Fisher; catalog no. MA1-23164), γ-H2AX (Millipore; catalog no. 05-636), normal mouse IgG (Santa Cruz; catalog no. SC-2025), bromodeoxyuridine (BrdU) (Abcam, Inc.; catalog no. Ab-8039), and anti-DNA-RNA hybrid antibody, clone S9.6 (Millipore; catalog no. MABE1095).

### Southern blot analysis.

Southern blot analysis was performed as previously described (16). Southern blots were stripped using Southern strip buffer (0.1× SSC [1× SSC is 0.15 M NaCl plus 0.015 M sodium citrate] and 0.5× SDS). The probed membrane was placed inside a hybridization system, and 50 ml of Southern strip buffer was added. The membrane was incubated with rotating at 95°C for 20 min twice. Following the second wash, 50 ml of 2× SSC was added to the glass cylinder and the cylinder was allowed to cool to room temperature. Once the cylinder had reached room temperature, hybridization buffer was added and probing and hybridization were performed as described above.

### PFGE Southern analysis.

Pulsed-field gel electrophoresis was performed using a BioRad Chef DR-II system at 120° and 4 V/cm^2^ with a switch time of 0.5 to 10 s over 15 h at 14°C, and the reaction mixture was subsequently probed with HPV 31 or was stripped and probed with an Alu sequence. The product of the Southern blotting was transferred using a GE Turbo blotter capillary transfer system. All other procedures used are outlined above.

### Double-strand break fluorescence *in situ* hybridization/comet-FISH.

HFKs, HFK 31 cells, or CIN612-9E cells were seeded into six-well plates for both differentiated and undifferentiated samples. Following differentiation, cells were treated with Versene to remove feeder fibroblasts and trypsinized and counted. Comet assays were performed using the Trevigen Comet assay (ES II system) per the manufacturer’s instructions through the electrophoresis step. Following the electrophoresis, the DBD-FISH assay was performed using a modified version of the Gosálvez protocol. After electrophoresis, reaction mixtures were washed in a Coplin jar in isotonic saline solution (0.9% NaCl at 4°C) for 2 min. Alkaline unwinding solution (0.03 M NaOH, pH 12.2) was freshly prepared and chilled to 4°C. Before the electrophoresis was performed, the alkaline unwinding solution was placed in a plastic tray on ice and allowed to reach approximately 7°C. After a 2-min wash in isotonic saline solution, the slides were placed in the alkaline unwinding solution for 2.5 min while being protected from light, followed by a 5-min wash in neutralizing solution (0.4 M Tris-HCl, pH 7.5). Next, the slides were transferred to TBE buffer (0.09 M Tris-borate, 0.002 M EDTA, pH 7.5) for 2 min. This was followed by 2 min (each) in 70%, 90%, and 100% ethanol baths. Slides were dried for 10 min in a slide dryer at 37°C. After drying, the biotinylated HPV 31 probe (Enzo) was denatured at 70° after being diluted 1:1 with FISH hybridization buffer (Empire Genomics) and subsequently chilled on ice for 3 min. A 15-μl volume corresponding to each condition was placed on each CometSlide, Parafilm was placed on top, and the slides were incubated in a humidity chamber for 72 h at room temperature. After hybridization, samples were incubated twice in 50% formamide–2× SSC at room temperature for 5 min, followed by two washes in 2× SSC for 3 min, followed by a 5-min wash in antibody washing solution (4× SSC, 0.1% Triton X-100, pH 7). Tyramide signal amplification was performed using TSA kit no. 22 (Life Technologies, Inc.) per the manufacturer’s instructions or Tyramide Super Boost 488. Samples were stained with DAPI and mounted in Gelvatol. Comets were visualized using a Zeiss Axioscope.

### Comet-EdU.

HFKs, HFK 31 cells, or CIN612-9E cells were seeded into six-well plates for both differentiated and undifferentiated samples. The morning of the assay, cells were supplemented with the 10 μM EdU supplied in a Click-iT EdU Alexa Fluor 594 imaging kit (Life Tech). Cells were treated with Versene to remove feeder fibroblasts and trypsinized and counted. Comet assays were performed using the Trevigen Comet assay (ES II system) per the manufacturer’s instructions. EdU was labeled with a fluor using a Click-iT EdU Alexa Fluor 594 imaging kit per the manufacturer’s instructions followed by total DNA staining and mounting in Gelvatol. Comets were visualized using a Zeiss Axioscope.

### T4 DNA ligase assay.

Cells were seeded onto coverslips and differentiated if required. When ready, cells were treated with 2 ml of PBS–4% methanol-free formaldehyde. PBS–0.1% Triton X-100 (PBT) was used as the permeabilization buffer, and cells were subsequently blocked in normal goat serum (Life Tech) with 0.1% Triton X-100 (NGS + T). Cells were rinsed in PBS three times for 5 min and were incubated in 100 μl of preincubation of solution (15% polyethylene glycol [PEG 8000], 66 mM Tris-HCl [pH 7.5], 5 mM MgCl_2_, 1 mM dithioerythritol, 1 mM ATP) for 30 min in a humidity chamber. The preincubation solution was removed, and a ligase reaction mix (15% PEG 8000, 1× T4 DNA ligase buffer, 750 ng of the biotin-labeled hairpin probe 5′-CGCCTAGACGTCGTCTAGCGCA-3′, 10 U T4 DNA ligase) (20 μl per sample) was applied for 18 h at room temperature in a humidity chamber. The next day, the coverslips were washed three times in distilled water for 10 min and then incubated in streptavidin-Texas Red conjugate diluted 1:500 in sodium bicarbonate buffer (50 mM NaHCO_3_, 15 mM NaCl [pH 8.2]) for 60 min at room temperature in a humidity chamber. Coverslips were then washed in distilled water three times for 10 min each time and mounted in Gelvatol. Coverslips were visualized using a Zeiss Axioscope and analyzed using FIJI software.

### Hydroxyurea and etoposide treatment.

Hydroxyurea (AbCam) was diluted in water at 100 mM and filter sterilized. Etoposide (Sigma) was diluted to 10 mM in dimethyl sulfoxide (DMSO). HU or etoposide was added every 48 h for undifferentiated cells and every 48 h upon addition of high levels of calcium for differentiated cells.

### Neutral comet assay.

HFKs, HFK 31 cells, or CIN612-9E cells were seeded into six-well plates for both differentiated and undifferentiated samples. Following differentiation, cells were treated with Versene to remove feeder fibroblasts and trypsinized and counted. Comet assays were performed using the Trevigen Comet assay (ES II system) per the manufacturer’s instructions. Comets were visualized using a Zeiss Axioscope, and percent tail DNA was quantitated programmatically using the open source software plugin OpenComet for FIJI.

### Immunofluorescence.

Cells were seeded onto coverslips and differentiated if required. When ready, cells were treated with 2 ml of PBS–4% methanol-free formaldehyde. PBS–0.1% Triton X-100 (PBT) was used as the permeabilization buffer, and cells were subsequently blocked in normal goat serum with 0.1% Triton X-100 (Life Tech). Primary antibodies were added to the blocking buffer at 1:50 or 1:100, and slides were incubated at 37°C for 1 h. Washes were performed with PBT. Secondary antibodies were added to blocking buffer, and slides were incubated for 45 min at 37°C. Slides were mounted using Gelvatol and DAPI and visualized using an epifluorescence microscope.

### Chromatin immunoprecipitation.

Chromatin immunoprecipitations were performed using HFKs, CIN612 cells, or stable HFK 31 cells in the presence or absence of HU. Cells were cultured as described above. Chromatin immunoprecipitation was performed using γ-H2AX (Millipore; catalog no. 05-636), RAD51 (Millipore; catalog no. 05-530-I), BRCA1 (Thermo Fisher; catalog no. MA1-23164), SMC-1 (Abcam, Inc.; catalog no. ab9262), and normal mouse IgG antibody (Santa Cruz; catalog no. SC-2025). Protocol was performed as previously described (37).

Real-time PCR was performed with a LightCycler 480 system (Roche) using HPV 31 URR primers, Alu primers, or HPV 31 L2 primers as follows: URR Forward, 5′-AAC|TGC|CAA|GGT|TGT|GTC|ATG|C-3′; URR Reverse, 5′-TGG|CGT|CTG|TAG|GTT|TGC|AC-3′; Alu Forward, 5′-ACG|AGG|TCA|GGA|GAT|CGA|GA-3′; Alu Reverse, 5′-CTC|AGC|CTC|CCA|AGT|AGC|TG-3′; L2 Forward, 5′-TTT|GGT|GGG|TTG|GGT|ATT|GG-3′; L2 Reverse, 5′-GTA|GGA|GGC|TGC|AAT|ACA|GAT|G-3′. All primers were determined to have similar levels of efficiency based on the methods previously described by Livak et al. (38).

### R-loop DNA-RNA hybrid immunoprecipitation (DRiP).

Total DNA lysates were collected as previously described and quantitated. DNA (25 to 50 μg) was digested with 1 U of mung bean nuclease (NEB) for 30 min at 37°C, and samples were sonicated as described above. Protein-G Dynabeads were precleared as described above and incubated all day with 2 μg/ml of anti-DNA/RNA hybrid s9.6 antibody, followed by addition of 100 μl of each sample in IP buffer as described above. The remainder of the ChIP analysis used the ChIP protocol described above. Real-time PCR was performed with a LightCycler 480 system (Roche) and the following HPV 31 URR primers and Alu primers: URR Forward, 5′-AAC|TGC|CAA|GGT|TGT|GTC|ATG|C-3′; URR Reverse, 5′-TGG|CGT|CTG|TAG|GTT|TGC|AC-3′; Alu Forward, 5′-ACG|AGG|TCA|GGA|GAT|CGA|GA-3′; Alu Reverse, 5′-CTC|AGC|CTC|CCA|AGT|AGC|TG-3′. All primers were determined to have similar levels of efficiency based on the methods previously described by Livak et al. (38).
