# Supplementary figures and images for: Human Papillomaviruses Preferentially Recruit DNA Repair Factors to Viral Genomes for Rapid Repair and Amplification
Source: mBio. 2018 Feb 13;9(1):e00064-18. doi: 10.1128/mBio.00064-18 (PMC5821098; doi:10.1128/mBio.00064-18)

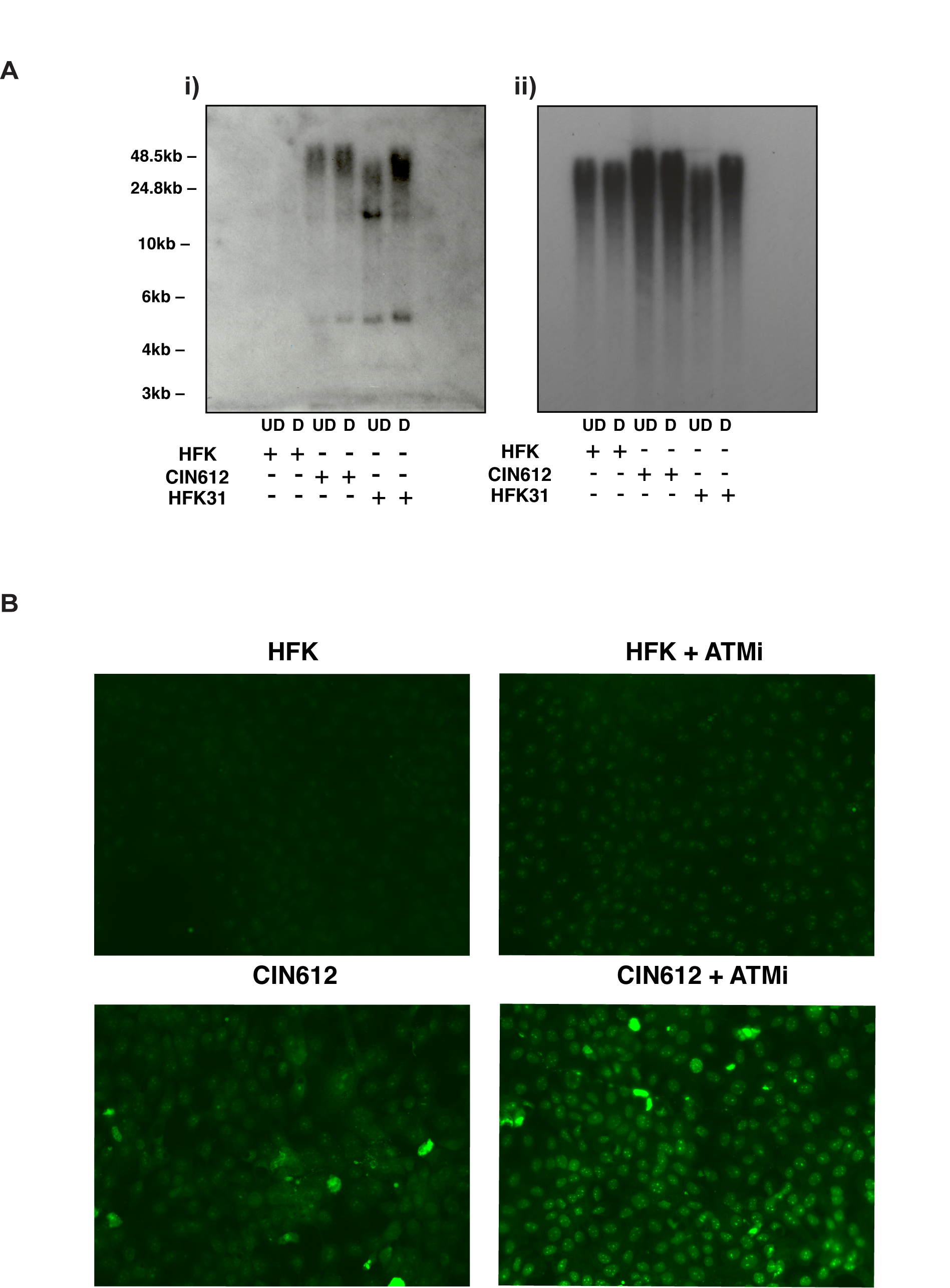

Supplement: FIG S1 [file mbo001183728sf1.tif]

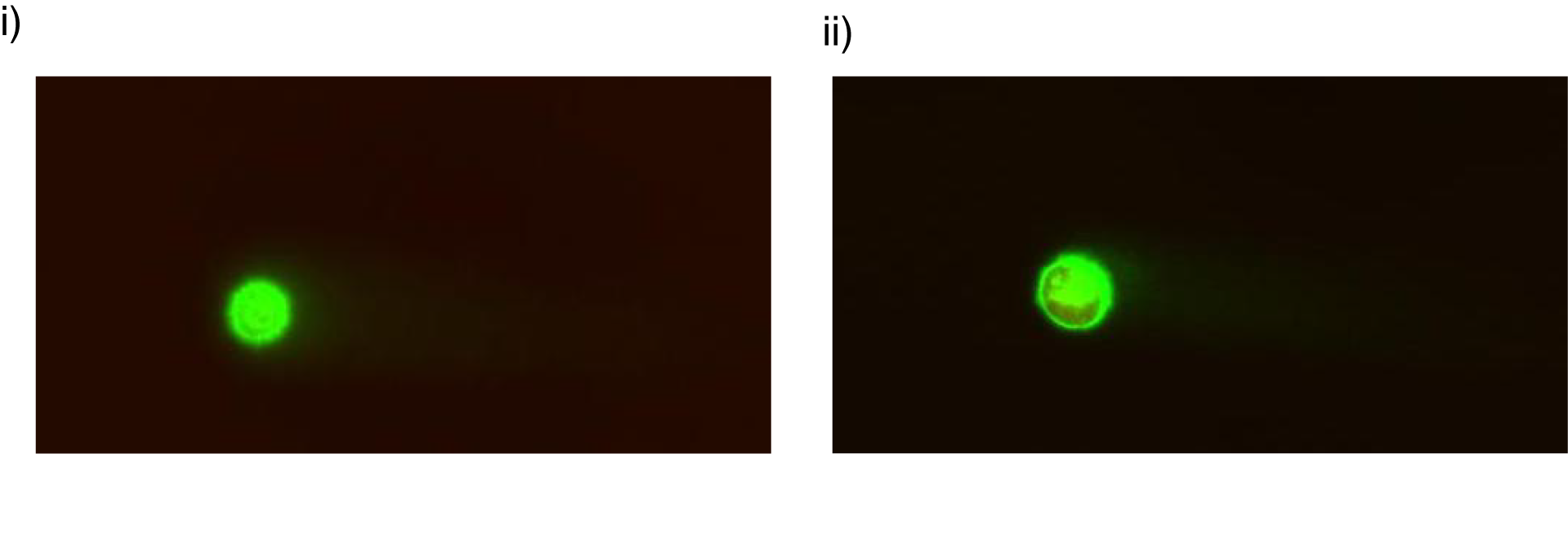

Supplement: FIG S2 [file mbo001183728sf2.tif]

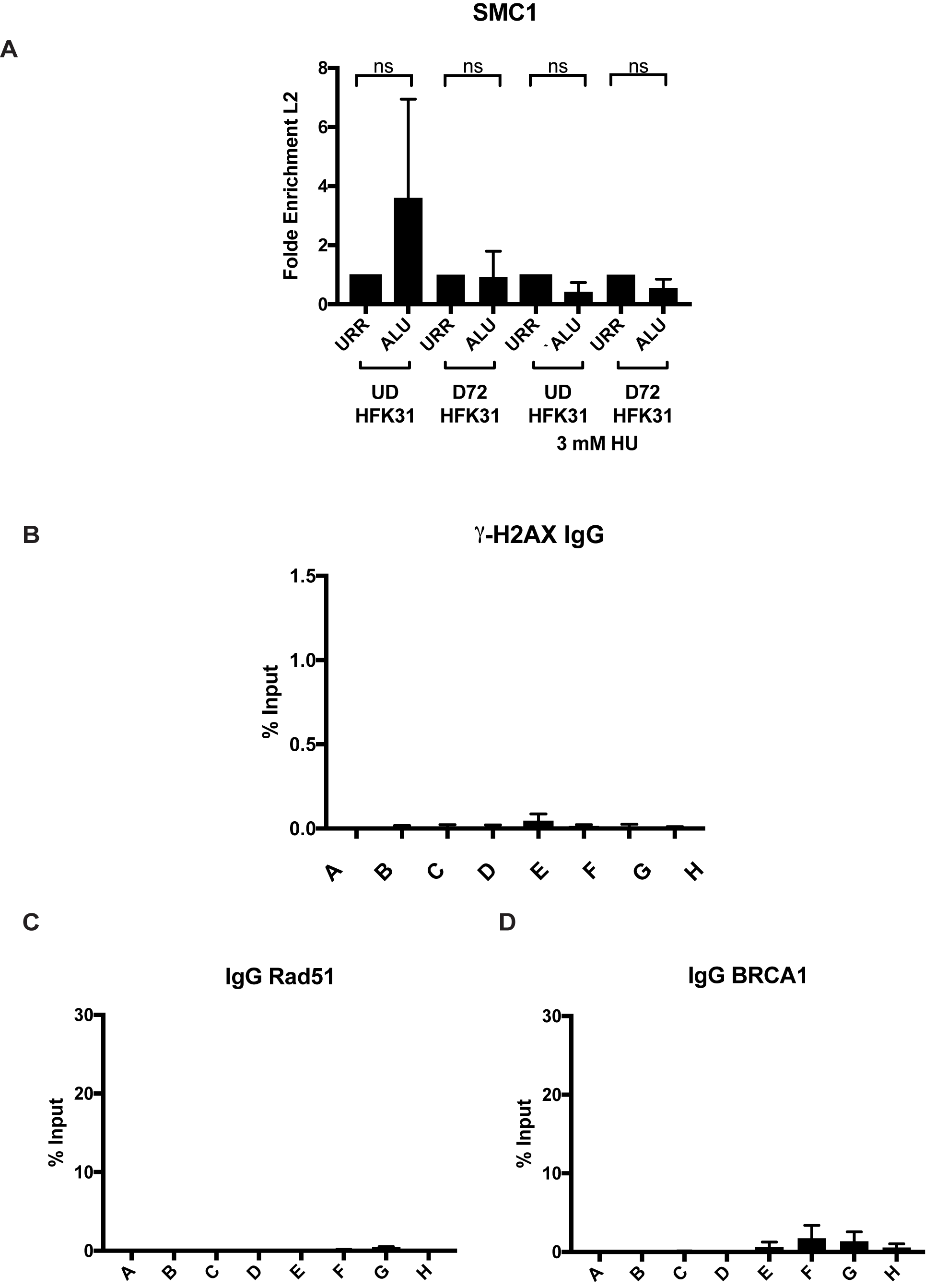

Supplement: FIG S3 [file mbo001183728sf3.tif]

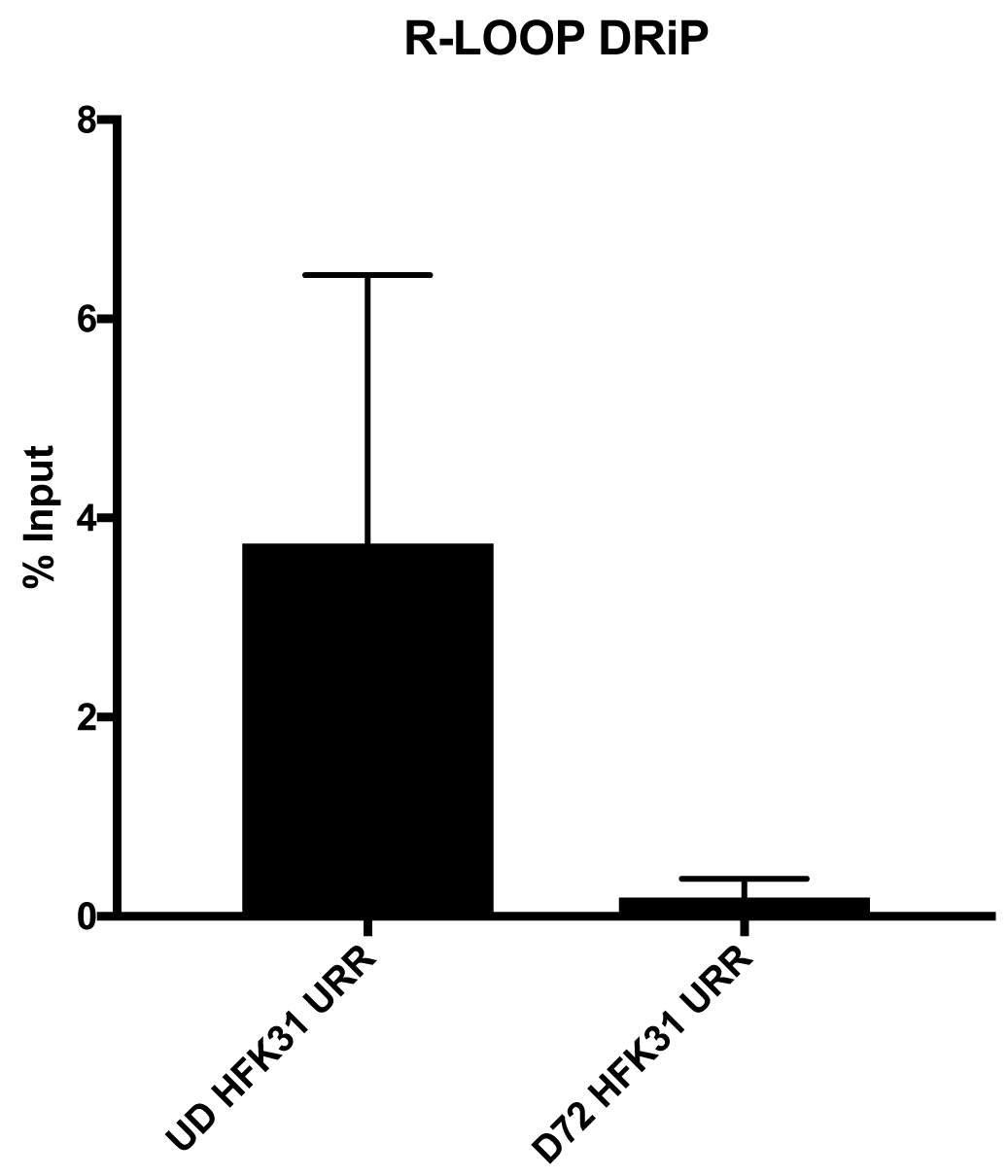

Supplement: FIG S4 [file mbo001183728sf4.tif]

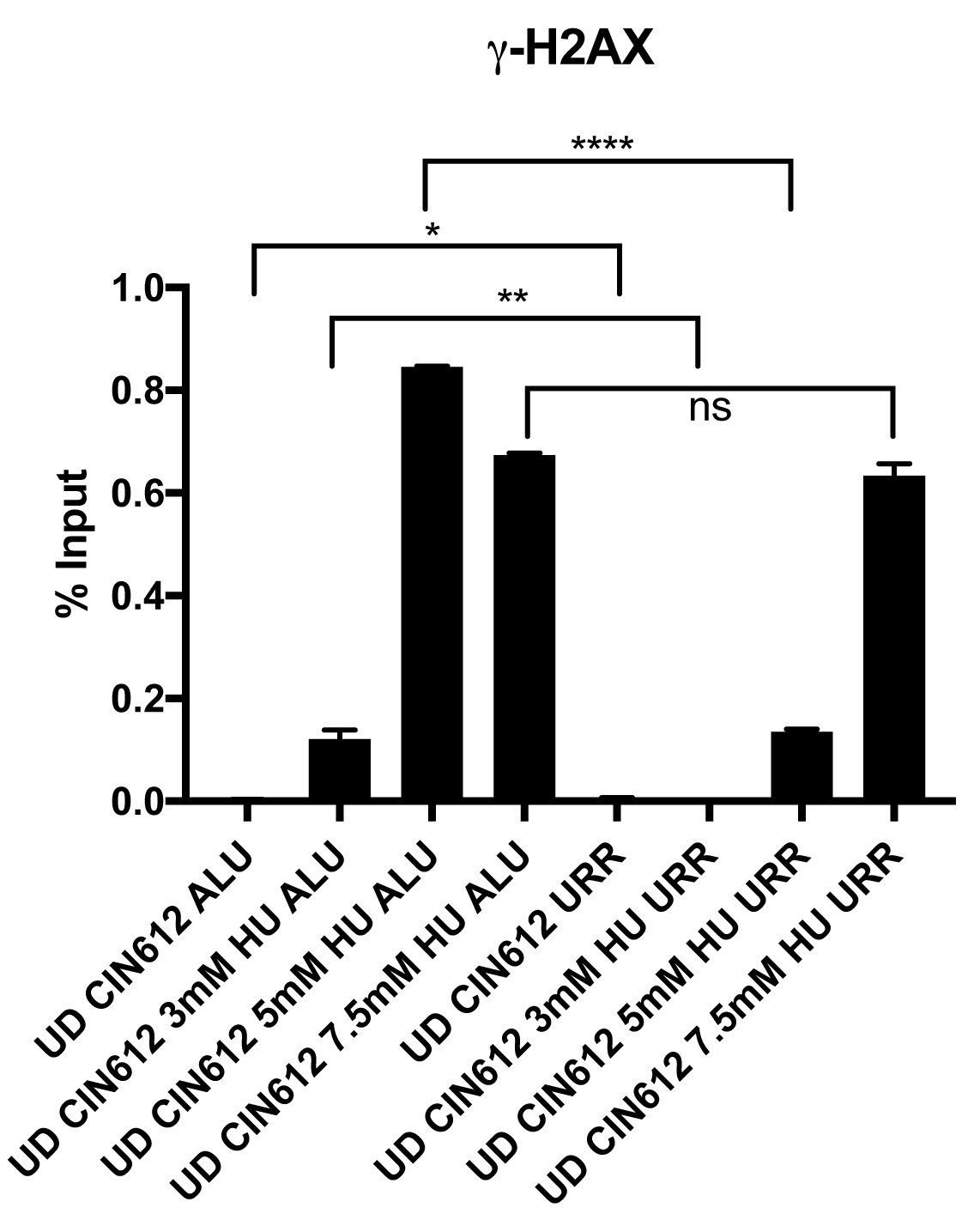

Supplement: FIG S5 [file mbo001183728sf5.tif]

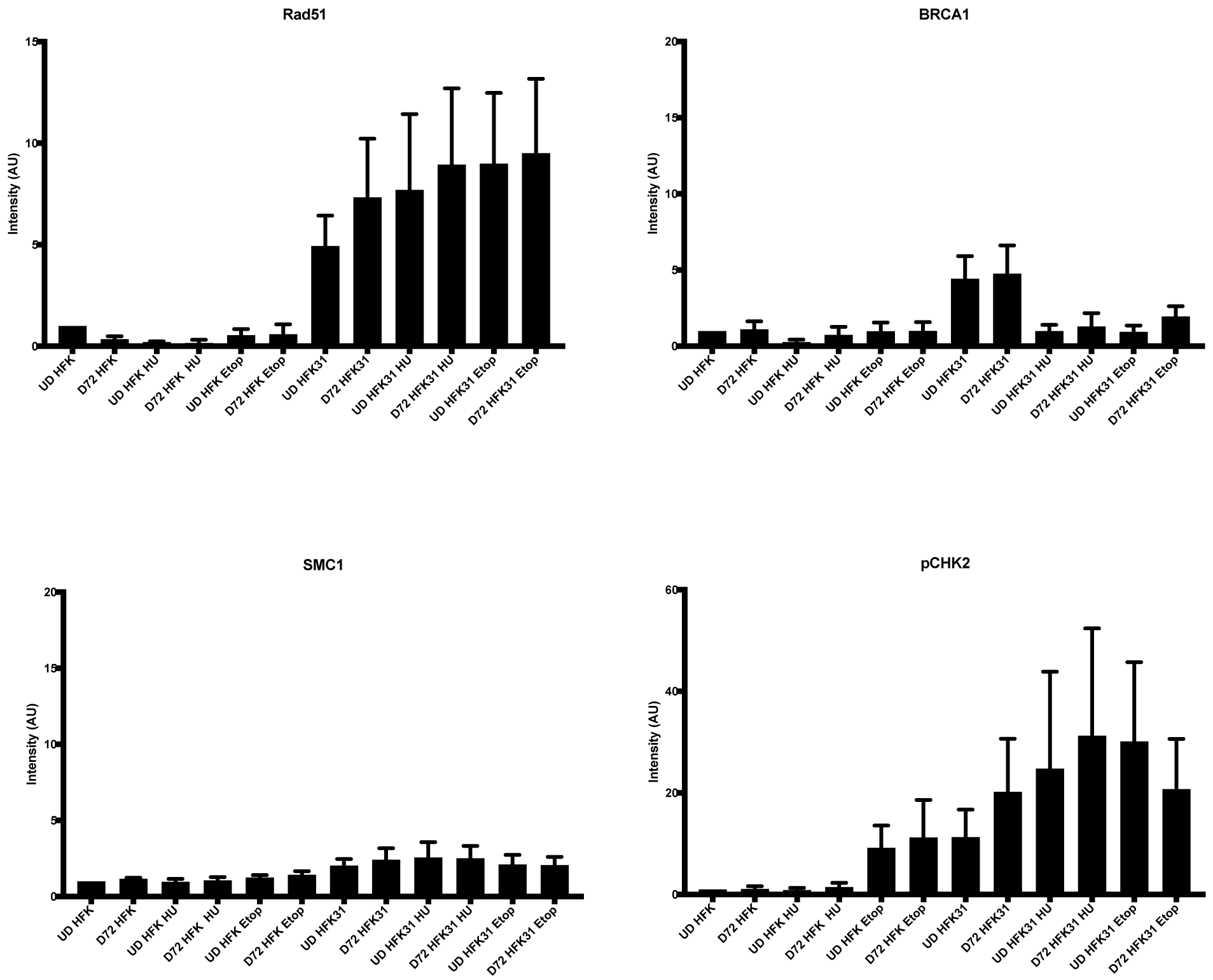

Supplement: FIG S6 [file mbo001183728sf6.tif]
